# Orthodontic intrusion of maxillary incisors: a 3D finite element method study

**DOI:** 10.1590/2177-6709.21.1.075-082.oar

**Published:** 2016

**Authors:** Armando Yukio Saga, Hiroshi Maruo, Marco André Argenta, Ivan Toshio Maruo, Orlando Motohiro Tanaka

**Affiliations:** 1 Professor, Pontifícia Universidade Católica do Paraná (PUC-PR), School of Health and Biosciences, Residency in Orthodontics, Curitiba, Paraná, Brazil; 2 Professor, Associação Brasileira de Odontologia (ABO-PG), Ponta Grossa, Paraná, Brazil; 3 Adjunct professor, Universidade Federal do Paraná (UFPR), Department of Civil Engineering, Graduate Program in Numerical Methods, Curitiba, Paraná, Brazil; 4 Professor, Pontifícia Universidade Católica do Paraná (PUC-PR), Residency in Orthodontics, and Associação Brasileira de Odontologia (ABO-PR), Curitiba, Paraná, Brazil; 5 Professor, Pontifícia Universidade Católica do Paraná (PUC-PR), School of Health and Biosciences, Graduate Dentistry Program in Orthodontics, Curitiba, Paraná, Brazil

**Keywords:** Orthodontics, Tooth intrusion, Finite element analysis

## Abstract

**Objective:**

In orthodontic treatment, intrusion movement of maxillary incisors is often necessary. Therefore, the objective of this investigation is to evaluate the initial distribution patterns and magnitude of compressive stress in the periodontal ligament (PDL) in a simulation of orthodontic intrusion of maxillary incisors, considering the points of force application.

**Methods:**

Anatomic 3D models reconstructed from cone-beam computed tomography scans were used to simulate maxillary incisors intrusion loading. The points of force application selected were: centered between central incisors brackets (LOAD 1); bilaterally between the brackets of central and lateral incisors (LOAD 2); bilaterally distal to the brackets of lateral incisors (LOAD 3); bilaterally 7 mm distal to the center of brackets of lateral incisors (LOAD 4).

**Results and Conclusions:**

Stress concentrated at the PDL apex region, irrespective of the point of orthodontic force application. The four load models showed distinct contour plots and compressive stress values over the midsagittal reference line. The contour plots of central and lateral incisors were not similar in the same load model. LOAD 3 resulted in more balanced compressive stress distribution.

## Abstract (pt)

**Objetivo:**

frequentemente, no tratamento ortodôntico, é necessário o movimento de intrusão dos incisivos superiores. Assim, o objetivo deste estudo é avaliar o padrão de distribuição inicial e magnitude das tensões compressivas no ligamento periodontal (LPD) na simulação da intrusão ortodôntica dos incisivos superiores, considerando os pontos de aplicação da força.

**Métodos:**

modelos anatômicos 3D reconstruídos a partir de tomografias computadorizadas de feixe cônico foram utilizados para simular os carregamentos da intrusão dos incisivos superiores. Os pontos eleitos para a aplicação das forças foram: centralizado entre os braquetes dos incisivos centrais (LOAD 1); bilateralmente, entre os braquetes dos incisivos centrais e laterais (LOAD 2); bilateralmente, distal aos braquetes dos incisivos laterais (LOAD 3); bilateralmente, 7mm distal ao centro dos braquetes dos incisivos laterais (LOAD 4).

**Resultados e Conclusões:**

as tensões concentraram-se na região apical do LPD, independentemente do ponto de aplicação da força ortodôntica; os quatro modelos de carregamento mostraram distribuição e valores de tensão compressiva distintos na linha mediana sagital de referência; os gráficos de distribuição das tensões não foram similares para os incisivos central e lateral no mesmo modelo de carregamento; o LOAD 3 resultou em uma distribuição mais equilibrada das tensões compressivas.

## INTRODUCTION

Regardless of genetic or treatment-related factors, maxillary incisors consistently feature more external apical root resorption (EARR) than any other tooth.^1^^,^ 2 With respect to the type of movement, intrusion movement appeared to be the most predictive for EARR.^3^^,^ 4 ^,^ 5 However, frequently, in orthodontic treatment, intrusion movement of an entire segment consisting of four maxillary incisors is necessary, as in cases of deep overbite correction.

With tridimensional (3D) numeric computer analysis, such as the finite element analysis (FEA), valuable information can be obtained, since various orthodontic clinical conditions can be simulated, and stress distribution in the individual constituents of the periodontium can be evaluated qualitatively and quantitatively.^6^^,^ 7

Studies relating the distribution of compression stress in the periodontal ligament (PDL) to the intrusion of maxillary incisors are scarce and approached in nonclinical conditions, since an intrusive force coincident with the long axes of four maxillary incisors is impossible to obtain clinically.^8^^,^ 9

Therefore, the objective of this investigation is to evaluate the initial distribution patterns and magnitude of compressive stress in the PDL in a simulation of orthodontic intrusion of maxillary incisors, considering the points of force application.

## MATERIAL AND METHODS

A maxilla from a dry adult human skull was reconstructed based on cone-beam computed tomography scans (i-CAT^TM^, Imaging Sciences, Hatfield, PA, USA), yielding a stack of 256 slices with 0.25 mm thickness, converted into exportable DICOM files. 

The limits of the compact and trabecular layers of bone, enamel and dentin were determined by using digital edge detection technology. The edges were then used to generate the 3D geometry with commercial computer-aided design software (Simpleware^TM^, Innovation Centre, Exeter, United Kingdom). The generated solid was exported as STL file (Stereolithography CAD) extension to Solidworks^TM^ (Dessault Systèmes Solidworks Corp., Concord, MA, USA) in order to convert into bilinear nonuniform rational B-spline (NURBS). The 0.022-in standard nontorqued, nonangulated edgewise orthodontic brackets and a cross section of 0.021 x 0.025-in arch wire were also 3D modeled. A 0.25-mm gap between roots and alveolar bone socket surfaces was considered as the space of the PDL.

This file was exported to ANSYS^TM^ v12.1 (Swanson Analysis System Inc., Canonsburg, PA, USA), the FEA solver software. The model was meshed by using tetrahedral elements of which quadratic shape allowed capturing the complex, curved surfaces in the modeling accuracy. The final model consisted of 322450 elements with edge length ranging from 0.25 mm to 1.50 mm and 603380 nodes.

Dental and bone material were assumed to be homogeneous, isotropic, and linearly elastic with specific Young's modulus and Poisson's ratios (Table 1).^10^^,^ 11 To represent the nonlinear mechanical behavior of the PDL, parameters of the hyperelastic instantaneous response were used (Table 2).^12^

**Table 1:** - Basic material properties of teeth and bone.

**Material**	**Young's modulus (MPa)**	**Poisson's ratios**
Enamel	84100^a^	0.20^a^
Dentin	18600^a^	0.31^a^
Compact bone	13800^a^	0.26^a^
Trabecular bone	345^a^	0.31^a^
Stainless steel	200000^b^	0.30^b^

a = Source: Jones et al.^(10)^; b = Source: Kojima and Fukui.^(11)^

**Table 2:** - Parameters of the hyperelastic instantaneous response of the PDL.

**C1 (MPa)**	**C2(MPa)**	**C3(MPa)**	**Kν (MPa)**	**β**
0.004	0.002	0.004	1000	3.5

**Figure 1:**
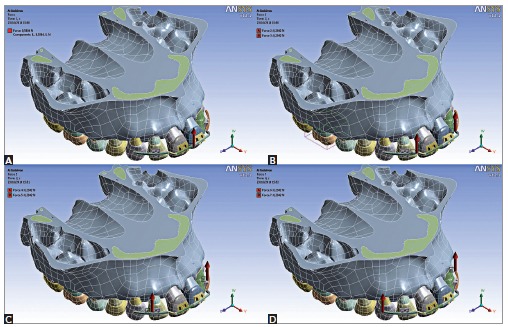
- Selected points of force application. A) LOAD 1; B) LOAD 2; C) LOAD 3; D) LOAD 4.

The points of force application were selected based on simulated clinical situations and considering points which the clinician usually would choose to intrude maxillary incisors. Thus, the points of force application were:

- LOAD 1: centered between central incisors brackets (Fig 1A).
- LOAD 2: bilaterally between central and lateral incisors brackets (Fig 1B).
- LOAD 3: bilaterally distal to lateral incisors brackets (Fig 1C).
- LOAD 4: bilaterally 7 mm distal to the center of lateral incisors brackets (Fig 1D).

To LOADs 2, 3 and 4, the points were selected also considering the approximate location of the center of resistance (CRes) of maxillary incisors. As a reference, it could lie apical of a point between the distal root side of the lateral incisor and the distal root side of the canine.^13^^-^ 16 No consensus has been reached in the literature in terms of the exact localization of CRes of maxillary incisors. Therefore, the points of force application varied in the 3D model in an attempt to approach, as close as possible, the center of resistance, which would result in more balanced stress distribution.

An intrusive force of 15 gf per tooth was applied to the model vertically, upwards and direct to the cross section of the 0.021 x 0.025-in archwire, since previous studies recommended a force magnitude varying from 10 to 20 gf per tooth, depending on the amount of periodontal support.^17^^,^ 18 It was imposed zero-displacement and zero-rotation boundary conditions on the nodes along the sliced maxilla in supra-apical horizontal plane.

XY scatter charts of stress values in a representative sagittal labial-apex-palatal (LAP) line were also considered, since the latter is located in the main plane to visualize maxillary incisors intrusion movement. The LAP representative line was defined by a reference line from the labial side of the PDL alveolar crest, going up to the apex to the palatal side of the alveolar crest, and matching, as close as possible, the midsagittal plane of the tooth.^5^^,^ 19 ^,^ 20 A total of 79 nodes were selected along this line for central incisors (relabeled from 1 to 79) and 88 for lateral incisors (relabeled from 1 to 88). Once the anatomical traits of the maxilla and teeth were clinically symmetrical, only teeth on the right side were considered for the XY scatter charts. 

**Figure 2:**
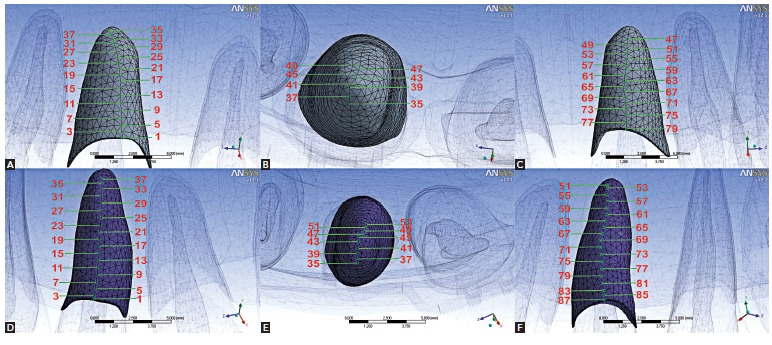
- Position of the odd numbered nodes in the LAP referential plane to right maxillary central incisor (A - labial view; B - apical view; C - palatal view) and to right maxillary lateral incisor (D - labial view; E - apical view; F - palatal view).

Figure 2 illustrates just the location of the odd numbered nodes for right maxillary central and lateral incisors in the LAP representative line. 

## RESULTS

The results are graphically demonstrated in two manners: contour plots and XY scatter charts representing nodal stress data in the PDL side of the PDL-socket bone interface, since stress in the PDL can be used to predict potential sites of bone remodeling.^21^

Records according to the minimum, mid and maximum principal stresses were obtained. In this study, minimum principal stress (MinPS) was equivalent to compressive stress. Hence, MinPS will be approached. 

### Contour plots

**Figure 3:**
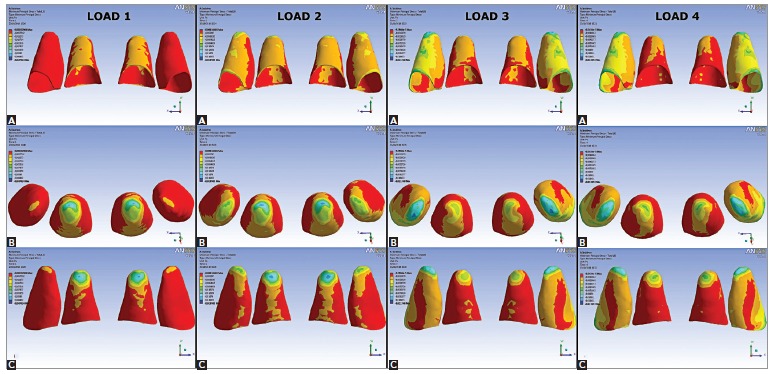
- MinPS (compression stress) distribution for maxillary incisors to LOADS 1, 2, 3 and 4: A) labial view; B) apical view; C) palatal view.

The contour plots for MinPS distribution are illustrated in Figure 3. Blue color shows areas of higher compression while red color refers to areas of lower compression. Throughout the simulation, the highest compressive areas were located at the apex.

In LOAD 1, the highest compression occurred mainly at the apex of central incisors. Overall, the labial side of the PDL presented a more extended compressive region than the palatal side. 

In LOAD 2, the highest compressive areas were observed at the apex of central incisors as well, but these areas shifted to mesial. Areas of compression were observed in the lateral incisors apex. Both central and lateral incisors exhibited higher compressive areas on the labial side in comparison to the palatal side, especially at the PDL labial margin of lateral incisors. 

In LOAD 3, the highest compressive areas shifted to lateral incisors apex. Compression areas in the PDL labial middle region and at the PDL labial margin were also present in these teeth. For central incisors, the palatal side of the apex exhibited higher compressive stress and almost the entire labial side of the PDL presented lower compression in comparison to the palatal side. 

Similarly to LOAD 3, in LOAD 4, the highest compression was observed at the lateral incisors apex as well. In the labial middle region and at the labial and mesial margin of lateral incisors PDL, compression was also present. Comparatively to LOAD 3, central incisors PDL labial side was less compressive. MinPS magnitudes (milliPascal, or mPa) for the four loading models are given inTable 3.

**Table 3:** - MinPS values (mPa) of the four loading simulation to maxillary right central and lateral incisors.

	**LOAD 1**	**LOAD 2**	**LOAD 3**	**LOAD 4**				
MinPS	Max	Min	Max	Min	Max	Min	Max	Min
Central incisor	-7.72	-40.14	-2.20	-15.30	-0.69	-3.80	-0.90	-5.06
Lateral incisor	-0.94	-5.30	-0.99	-8.49	-1.29	-9.89	-1.74	-9.57

Note: the more negative the value, the higher the compressive stress.

### MinPS XY scatter charts - Right maxillary central incisor (Fig 4).

In every loading model, node 45, located in the PDL apex, showed the highest compression; i.e., most negative values. In LOAD 1, on the labial side, higher compression in the PDL cervical third occurred comparatively to the palatal side. From node 25 (labial apical third) to node 35, a sharper compression increase occurred. Node 45, located at the PDL apex, presented the highest compression (-40.14 mPa). From node 45, compression descended steeply. The line between nodes 55 and 79 referred approximately to the cervical and middle thirds of the PDL palatal side. 

**Figure 4:**
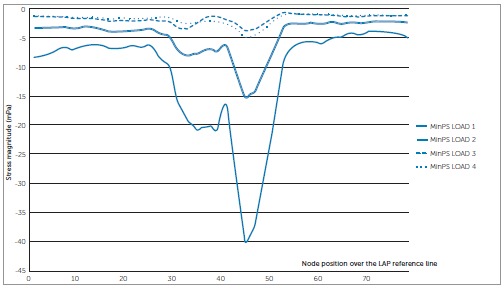
- MinPS (compression stress) scatter chart to the four loading simulations according to node position over the LAP reference line for the right maxillary central incisor.

LOAD 2 had a similar behavior to LOAD 1; however, in all nodes, less compression was observed; e.g., in node 45, MinPS was -15.30 mPa (2.62 less compression than in LOAD 1). Moreover, from node 1 to 25, on PDL labial side, compression was relatively constant.

LOADS 3 and 4 revealed the lowest compression (-3.80 mPa and -5.06 mPa, respectively) and variations of stress along the LAP reference line, showing the smallest differences between maximum and minimum values. Thus, they demonstrated a more balanced distribution of stress.

### MinPS XY scatter charts - Right maxillary lateral incisor (Fig 5)

Comparatively to central incisors, stress distribution over the LAP reference line was more irregular for lateral incisors, with more up and down variations. For LOAD 1, there was a tendency from node 1 to 44 towards a moderate compression decrease. Node 44 had the highest compression stress (-5.30 mPa). From this node until node 56, the decrease in compression stress was followed by a tendency towards stabilization of compression. For LOADs 2, 3 e 4, the graphic line behavior was similar. The PDL labial side demonstrated great nodal stress variations along the LAP reference line. Compression stress was observed in the PDL labial margin in LOAD 4 (-7.11 mPa), followed by LOAD 3 (-6.08 mPa) and 2 (-5.29 mPa). 

**Figure 5:**
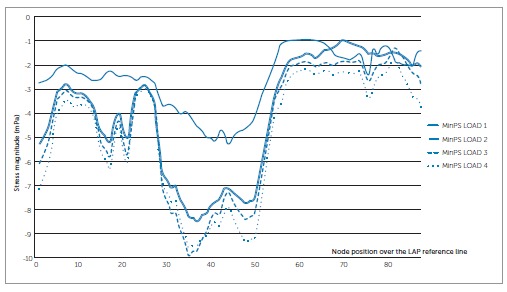
- MinPS (compression stress) scatter chart to the four loading simulations according to node position over the LAP reference line for the right maxillary lateral incisor.

In the PDL apex, the highest compression stress was observed at the apical area at node 35 to LOAD 4 (-9.57 mPa) and LOAD 3 (-9.89 mPa), at node 36 to LOAD 2 (-8.33 mPa) and at node 44 (-5.30 mPa) to LOAD 1. 

## DISCUSSION

FEA is a computer engineering numeric method that has allowed solution of biomechanical problems not involving live organisms. The 3D model of the maxilla and teeth obtained from a real anatomic skull and the discretization of individual components of bone (trabecular and cortical bone) and tooth (enamel and dentin) in this study render the results as closer as possible to a real situation. 

Clinically, intrusive forces have been traditional suspect in severe cases of root resorption.^4^^,^ 5 ^,^ 22 The present finite element model study showed that there was stress concentration at the PDL of the root apex. Shaw, Sameshima and Vu,^7^ as well as Parker and Harris,^5^ reported that intrusive movement and increase in incisor proclination were the most powerful predictors of EARR. Comparatively, LOADs 1 and 2 were mechanical configurations with the highest compression in the apical region of the PDL, and also had the greatest tendency towards buccal proclination of central incisors. Thus, from a biological and mechanical standpoint, they could be the least desirable points of force application for intrusion of maxillary incisors. Even though this study has demonstrated stress concentration in the apical region of the PDL, clinical studies^20^^,^ 23 showed no differences in the amount of root resorption between intrusion and other orthodontic movements, demonstrating that there are still other factors or variables to be explored. 

In addition, factors that alter the position of the CRes of four maxillary incisors are the shape of surrounding bone, root morphology, position of each tooth, and structure of the periodontal attachment.^9^^,^ 24 Since these factors will generally be different for each patient, the location of the CRes of anterior arch segments in these patients will also be different. 

*In vitro* studies using different methods^13^^,^ 15 ^,^ 25 ^,^ 26 showed that the CRes of the four incisors lies 8-10 mm apical and 5-7 mm distal to lateral incisors. A more anterior location of the point of force application causes flaring, whereas a more posterior location will cause uprighting of anterior teeth. When the axial inclination of incisors is different, so is the location of the axes of resistance in relation to the position of incisors crowns. More flared incisors should have a more distal point of force application than retroclined incisors.^16^However, it is important to report that the 3D model produced had just a slight maxillary incisor protrusion, and this fact might have influenced the results in relation to stress distribution. Even if there is no common center or axis of resistance to the four incisors, it is necessary to determine a line of action of force that promotes a more balanced stress distribution.

LOAD 1 and LOAD 2 mechanical configurations, especially LOAD 1, showed a strong tendency towards proclination of maxillary central incisors; whereas for LOAD 3 and LOAD 4, the orthodontic movement likely to occur would be intrusion with little or no protrusion, but with distal inclination of lateral incisors in LOAD 4.

Although LOAD 3 and LOAD 4 presented more balanced stress distribution, in agreement with Reimann et al,^25^ it is important to note that the central incisors are loaded with smaller force systems than lateral incisors. This, in turn, means that lateral incisors are loaded higher by the applied force system, which could compromise periodontal support, since lateral incisors usually have a smaller root surface area than central incisors.

As in most computer simulations of biological situations, the limitations of the study are mainly regarding material parameters. It must be stated that values reported in the literature differ significantly from each other, especially in terms of the PDL. These differences are due to experimental designs, large variation in the complexity and geometry of numerical models.^27^ Evidently the applied parameters did not reproduce perfectly the complex structure and behavior of dental, bone and PDL tissues. Nevertheless, it was assumed that this behavior idealization was suitable to describe theoretically the initial stress distribution of maxillary incisors orthodontic intrusion and that the results could be considered in clinical treatment planning.

Computed tomography scans and 3D reconstructions have become common examinations in orthodontic diagnosis. They allow individual determination of the most suitable points/axes of force application by computer modeling and numerical simulation. Thus, in the treatment of patients with complex problems, in which risks are greater, they may assist the implementation of a more effective orthodontic mechanics in order to obtain greater predictability of orthodontic movement with minimal side effects.

## CONCLUSIONS

Within the study methodology, it is possible to conclude the following:

- Stress is concentrated at the PDL apex region, irrespective of the point of orthodontic force application;
- The four load models showed distinct contour plots and compressive stress values over the LAP reference line;
- The contour plots of central and lateral incisors were not similar in the same load model;
- LOAD 3 resulted in more balanced compressive stress distribution.
